# A cohort autopsy study defines COVID-19 systemic pathogenesis

**DOI:** 10.1038/s41422-021-00523-8

**Published:** 2021-06-16

**Authors:** Xiao-Hong Yao, Tao Luo, Yu Shi, Zhi-Cheng He, Rui Tang, Pei-Pei Zhang, Jun Cai, Xiang-Dong Zhou, Dong-Po Jiang, Xiao-Chun Fei, Xue-Quan Huang, Lei Zhao, Heng Zhang, Hai-Bo Wu, Yong Ren, Zhen-Hua Liu, Hua-Rong Zhang, Cong Chen, Wen-Juan Fu, Heng Li, Xin-Yi Xia, Rong Chen, Yan Wang, Xin-Dong Liu, Chang-Lin Yin, Ze-Xuan Yan, Juan Wang, Rui Jing, Tai-Sheng Li, Wei-Qin Li, Chao-Fu Wang, Yan-Qing Ding, Qing Mao, Ding-Yu Zhang, Shu-Yang Zhang, Yi-Fang Ping, Xiu-Wu Bian

**Affiliations:** 1 Institute of Pathology, Southwest Hospital, Third Military Medical University (Army Medical University), Chongqing, China; 2 Key Laboratory of Tumor Immunopathology, Ministry of Education of China, Chongqing, China; 3 Department of Pathology, the First Hospital Affiliated to University of Science & Technology of China, Hefei, Anhui China; 4 Department of Pathology, Ruijin Hospital, School of Medicine, Shanghai Jiaotong University, Shanghai, China; 5 Department of Pathology, School of Medicine, Shanghai Jiaotong University, Shanghai, China; 6 Department of Pulmonary & Critical Care Medicine, Southwest Hospital, Third Military Medical University (Army Medical University), Chongqing, China; 7 Wound Trauma Medical Center, State Key Laboratory of Trauma, Daping Hospital, Third Military Medical University, Chongqing, China; 8 Department of Vascular Surgery, Southwest Hospital, Third Military Medical University (Army Medical University), Chongqing, China; 9 Department of Pathology, General Hospital of Central Theater Command of PLA, Wuhan, Hubei China; 10 Department of Ultrasound, Ruijin Hospital, School of Medicine, Shanghai Jiaotong University, Shanghai, China; 11 Institute of Laboratory Medicine, Jinling Hospital, School of Medicine, Nanjing University, the First School of Clinical Medicine, Southern Medical University, Nanjing, Jiangsu China; 12 Wuhan Jinyintan Hospital (Wuhan Hospital for Infectious Diseases), Wuhan, Hubei China; 13 Department of Critical Care Medicine, Southwest Hospital, Third Military Medical University (Army Medical University), Chongqing, China; 14 Emergency Department and Clinical Skills Training Center, Southwest Hospital, Third Military Medical University (Army Medical University), Chongqing, China; 15 Department of Pathology and Pathophysiology, School of Medicine, Zhejiang University, Hangzhou, Zhejiang China; 16 Department of Infectious Diseases, Peking Union Medical College Hospital, Beijing, China; 17 Department of Critical Care Medicine, PLA Key Laboratory of Emergency and Critical Care Research, Jinling Hospital, Nanjing University, Nanjing, Jiangsu China; 18 Department of Pathology, Nan Fang Hospital, Southern Medical University, Guangzhou, Guangdong China; 19 Department of Infectious Diseases, Southwest Hospital, Third Military Medical University (Army Medical University), Chongqing, China; 20 Peking Union Medical College Hospital, Beijing, China

**Keywords:** Mechanisms of disease, Immunology

## Abstract

Severe COVID-19 disease caused by SARS-CoV-2 is frequently accompanied by dysfunction of the lungs and extrapulmonary organs. However, the organotropism of SARS-CoV-2 and the port of virus entry for systemic dissemination remain largely unknown. We profiled 26 COVID-19 autopsy cases from four cohorts in Wuhan, China, and determined the systemic distribution of SARS-CoV-2. SARS-CoV-2 was detected in the lungs and multiple extrapulmonary organs of critically ill COVID-19 patients up to 67 days after symptom onset. Based on organotropism and pathological features of the patients, COVID-19 was divided into viral intrapulmonary and systemic subtypes. In patients with systemic viral distribution, SARS-CoV-2 was detected in monocytes, macrophages, and vascular endothelia at blood–air barrier, blood–testis barrier, and filtration barrier. Critically ill patients with long disease duration showed decreased pulmonary cell proliferation, reduced viral RNA, and marked fibrosis in the lungs. Permanent SARS-CoV-2 presence and tissue injuries in the lungs and extrapulmonary organs suggest direct viral invasion as a mechanism of pathogenicity in critically ill patients. SARS-CoV-2 may hijack monocytes, macrophages, and vascular endothelia at physiological barriers as the ports of entry for systemic dissemination. Our study thus delineates systemic pathological features of SARS-CoV-2 infection, which sheds light on the development of novel COVID-19 treatment.

## Introduction

Severe acute respiratory syndrome coronavirus 2 (SARS-CoV-2), a novel beta-coronavirus, has given rise to a global pandemic. In comparison with the other two coronavirus strains responsible for Middle Eastern respiratory syndrome (MERS) and severe acute respiratory syndrome (SARS), SARS-CoV-2 is associated with lower mortality but with high transmission efficiency.^1^ A large body of clinical data from tertiary referral centers showed that although most patients with COVID-19 show no symptoms or mild symptoms, the elders with underlying diseases are prone to developing severe or critical disease condition with high mortality.^2–4^ In addition to respiratory manifestations, extra-pulmonary involvements are increasingly recognized, including dysfunctions of the immune system, cardiovascular system, gastrointestinal system, and neurological system.^3,4^ However, regarding the pathogenicity of COVID-19, it remains unclear whether the systemic pathological changes are driven by local viral attack, the maladaptive immune responses, or the consequences of therapeutics.

Autopsies of COVID-19 deceased patients and experimental animal studies have provided insights into the fundamental pathological changes in the lungs and multiple extrapulmonary organs.^5–12^ We and others have found that the lungs are the most affected organ by SARS-CoV-2, showing diffuse alveolar damage, exudation, interstitial fibrosis, extensive infiltration of immune cells, including dysfunctional alveolar macrophages, and abundant inflammatory factors.^7,13,14^ Extrapulmonary organs exhibited different extent of tissue injuries and inflammatory responses. In particular, the lymphatic organs such as the spleen and lymph nodes contained reduced lymphocytes but increased macrophages.^7^ These findings provide the pathological basis for severe hypoxia (acute respiratory distress syndrome (ARDS)) and immune malfunction in critically ill patients with COVID-19. However, information about the tissue tropism of SARS-CoV-2 and the port of virus entry responsible for systemic dissemination is sparse. A recent study provides evidence for SARS-CoV-2 entry into the nervous system by crossing the neural-mucosal interface in olfactory mucosa.^15^ However, it remains unknown whether SARS-CoV-2 penetrates through physical barriers into target organs.

It is believed that definitive detection of SARS-CoV-2 spike or nucleocapsid protein by immunohistochemical (IHC) staining should confirm in situ viral presence in the lungs and extrapulmonary organs, thereby establishing direct evidence of viral infection of target organs.^16–19^ In this study, we profiled SARS-CoV-2 organotropism using 26 autopsy cases from four cohorts in Wuhan, China, and evaluated virus-associated organ injuries. We classified COVID-19 into two pathological subtypes as intrapulmonary infection and systemic infection respectively. Our study delineates SARS-CoV-2 organotropism and pathogenicity, which provides a better understanding of the mechanisms of viral infection and novel therapeutic approaches to minimizing the systemic dissemination of the virus.

## Results

### Characteristics of the cohort

**Fig. 1:**
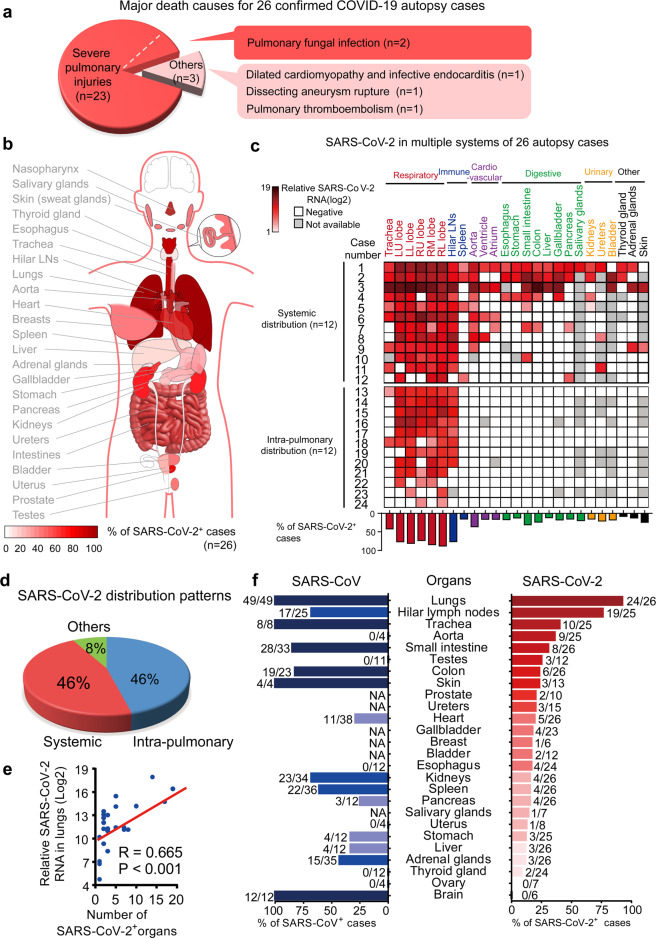
Profiling of SARS-CoV-2 organotropism in COVID-19 patients. **a** Major death causes for the 26 autopsy COVID-19 cases. The major death causes were severe pulmonary injuries (*n* = 23), including COVID-19-related respiratory failure without (*n* = 21) or with (*n* = 2) pulmonary fungal infection. The major death causes for other three cases were pulmonary thromboembolism, dissecting aneurysm rupture, and cardiovascular disorders, respectively. **b** Schematic model for SARS-CoV-2 organ tropism. LNs, Lymph nodes. **c** Heatmap showing SARS-CoV-2 distribution groups and viral RNA (Log2) in postmortem organs in 26 autopsy cases with COVID-19. LU, left upper; LL, left lower; RU, right upper; RM; right middle; RL, right lower. **d** Percentage of COVID-19 autopsy cases in three groups of SARS-CoV-2 distribution. **e** The correlation between SARS-CoV-2 viral RNA in the lungs and the number of SARS-CoV-2-positive organs. **f** Comparison of viral infection rate between SARS-CoV-2 based on the current autopsy study and SARS-CoV in the literature in postmortem organs from patients with COVID-19 and SARS.

All 26 autopsy cases used in this study were deceased patients (median 67.5-year-old) and met the diagnostic criteria for critically ill status of COVID-19. The presence of SARS-CoV-2 in all cases was confirmed by PCR tests, IHC staining of viral spike or nucleoprotein, or electron microscopy. The basic information, disease duration, clinical manifestations, and treatment regimens for 26 cadaver donors were summarized in Tables 1, 2; Supplementary information, Fig. S1. Twenty cases (76.9%) were diagnosed with underlying diseases, including chronic pulmonary diseases (23.1%), chronic cardiovascular diseases (38.5%), and hypertension (34.6%). The median duration from COVID-19 symptom onset to death was 38.5 days. The major death causes for our COVID-19 autopsy cases included severe pulmonary injuries (COVID-19-related respiratory failure with/without pulmonary fungal infection), pulmonary thromboembolism, dissecting aneurysm rupture, and cardiovascular disorders (Fig. 1a and Table 1).

**Table 1:** Major death causes of 26 COVID-19 autopsy cases diagnosed with SARS-CoV-2 infection.

Case ID	Gender	Age	Survival since symptom onset (days)	Death causes
Case 1	M	77	65	Respiratory failure related to SARS-CoV-2
Case 2	M	76	29	1. Respiratory failure related to SARS-CoV-2 2. Secondary bacterial infection
Case 3	F	73	36	1. Respiratory failure related to SARS-CoV-2 2. Secondary bacterial infection
Case 4	M	87	15	1. Respiratory failure related to pulmonary fungal infection 2. SARS-CoV-2 related pneumonia
Case 5	M	70	46	1. Respiratory failure related to SARS-CoV-2 2. Multiple organ hemorrhage
Case 6	M	64	30	Respiratory failure related to SARS-CoV-2
Case 7	F	57	35	1. Respiratory failure related to SARS-CoV-2 2. Secondary infection
Case 8	F	74	30	1. Pulmonary thromboembolism 2. Respiratory failure related to SARS-CoV-2
Case 9	F	66	37	1. Respiratory failure related to SARS-CoV-2 2. Secondary bacterial infection
Case 10	F	53	28	Respiratory failure related to SARS-CoV-2
Case 11	M	68	45	1. Respiratory failure caused by SARS-CoV-2 2. Multiple organ thromboembolism
Case 12	M	88	20	1. Respiratory failure related to SARS-CoV-2
Case 13	F	87	46	1. Respiratory failure related to SARS-CoV-2 2. Pulmonary thromboembolism
Case 14	M	62	22	Respiratory failure related to SARS-CoV-2
Case 15	F	56	42	1. Respiratory failure related to SARS-CoV-2 2. Pulmonary hyaline thromboembolism
Case 16	F	84	36	Respiratory failure related to SARS-CoV-2
Case 17	M	81	57	1. Respiratory failure related to SARS-CoV-2 2. Pulmonary thromboembolism
Case 18	M	59	65	Respiratory failure related to SARS-CoV-2
Case 19	F	60	37	1. Hemorrhagic shock due to dissecting aneurysm rupture 2. Multiple organ hemorrhage
Case 20	M	67	51	1. Respiratory failure related to SARS-CoV-2 2. Secondary infection
Case 21	M	68	52	Respiratory failure related to SARS-CoV-2
Case 22	F	80	62	Respiratory failure related to SARS-CoV-2
Case 23	F	63	40	1. Respiratory failure related to SARS-CoV-2 2. Pulmonary hemorrhage
Case 24	F	59	67	1. Respiratory failure related to SARS-CoV-2 2. Pulmonary hemorrhage
Case 25	F	65	34	Heart failure related to dilated cardiomyopathy and infective endocarditis
Case 26	M	70	56	1. Respiratory failure due to pulmonary fungal infection 2. SARS-CoV-2 related pneumonia

SARS-CoV-2, severe acute respiratory syndrome coronavirus 2; F, female; M, male.

**Table 2:** Clinical characteristics of 26 autopsy cases with SARS-CoV-2 infection.

	Systemic distribution (*n* = 12)	Intrapulmonary distribution (*n* = 12)	Others (*n* = 2)	All autopsy cases (*n* = 26)
Age, years	67.5 (3.5)	68.8 (11.1)	71.1 (11.5)	69.8 (10.3)
Gender				
Female	5 (41.7%)	7 (58.3%)	1 (50.0%)	13 (50.0%)
Male	7 (58.3%)	5 (41.7%)	1 (50.0%)	13 (50.0%)
Survival since the onset of symptoms, days	35.5 (28–45)	48.5 (37–62)	45 (34–56)	38.5 (30–52)
Hospitalization, days	21 (6–32)	29 (21–50)	21.5 (10–33)	26.5 (15–35)
ICU Hospitalization, days	14 (4–22)	23.5 (15–37)	3 (3)	20 (6–26)
Clinical symptoms				
Fever	11 (91.7%)	12 (100.0%)	1 (50.0%)	24 (92.3%)
Cough	11 (91.7%)	12 (100.0%)	1 (50.0%)	24 (92.3%)
Sputum production	6 (50.0%)	8 (66.7%)	1 (50.0%)	15 (57.7%)
Diarrhea	5 (41.7%)	7 (58.3%)	0 (0.0%)	12 (46.2%)
Malaise	4 (33.3%)	7 (58.3%)	2 (100.0%)	13 (50.0%)
Imaging features				
Bilateral ground-glass opacity	12 (100.0%)	12 (100.0%)	2 (100.0%)	26 (100.0%)
Bilateral pulmonary infiltration	12 (100.0%)	12 (100.0%)	1 (50.0%)	25 (96.2%)
Pleural effusion	10 (83.3%)	6 (50.0%)	1 (50.0%)	17 (65.4%)
Consolidation	12 (100.0%)	12 (100.0%)	0 (0.0%)	24 (92.3%)
White-cell count × 10^9^/L				
Median (IQR)	11.4 (7.6–14.4)	10.7 (7.2–13.8)	9.4 (5.4–13.5)	10.7 (7.5–14.1)
≥ 10	7 (58.3%)	7 (58.3%)	1 (50.0%)	15 (57.7%)
≤ 4	0 (0.0%)	0 (0.0%)	0 (0.0%)	0 (0.0%)
Lymphocyte count × 10^9^/L				
Median (IQR)	0.4 (0.3–0.6)	0.7 (0.5–1.0)	0.6 (0.1–1.2)	0.5 (0.3–1.0)
≤ 1.5	11 (91.7%)	12 (100.0%)	2 (100.0%)	25 (96.2%)
Monocyte count × 10^9^/L				
Median (IQR)	0.2 (0.2–0.4)	0.5 (0.3–0.7)	1.0	0.4 (0.2–0.7)
> 0.6	2 (16.7%)	4 (33.3%)	1 (50.0%)	7 (26.9%)
< 0.1	1 (8.3%)	0 (0.0%)	NA	1 (3.9%)
History				
Chronic pulmonary diseases	2 (16.7%)	3 (25.0%)	1 (50.0%)	6 (23.1%)
Chronic cardiac diseases	4 (33.3%)	5 (41.7%)	1 (50.0%)	10 (38.5%)
Chronic dialysis	0 (0.0%)	0 (0.0%)	0 (0.0%)	0 (0.0%)
Hypertension	4 (33.3%)	4 (33.3%)	1 (50.0%)	9 (34.6%)
Diabetes	2 (16.7%)	1 (8.3%)	1 (50.0%)	4 (15.4%)
Malignancy	2 (16.7%)	0 (0.0%)	0 (0.0%)	2 (7.7%)

Data are presented as *n* (%) or mean (SD) or median (IQR, interquartile range).

SARS-CoV-2, severe acute respiratory syndrome coronavirus 2; NA, not available.

### Overview of SARS-CoV-2 virus organotropism

We and others have identified the pathological changes of multiple organs through systemic autopsy examination of COVID-19 patients,^5–10^ although information about the duration of SARS-CoV-2 in target organs and its correlation with disease progression is sparse. Through systemic autopsy examination, we found that SARS-CoV-2 RNA, spike protein or virion-like particles existed in the lungs and multiple extrapulmonary organs in critically ill patients as long as 15–67 days after symptom onset. The SARS-CoV-2 viral RNA distributed in postmortem organs including those in the respiratory, digestive, genitourinary, cardiovascular, immune systems, endo/exocrine glands, and skin (Fig. 1b, c). The viral distribution patterns were categorized into three groups: systemic (12/26, 46.2%), intrapulmonary (12/26, 46.2%), and others (2/26, 7.7%) (Fig. 1b–d). Although COVID-19-related respiratory failure was present in both systemic and intrapulmonary distribution groups, cases in the systemic distribution group also exhibited increased probabilities of extrapulmonary organ failures (especially the failure of kidney, heart, and liver). Two cases (Cases 25 and 26) categorized into other distribution group did not show SARS-CoV-2 RNA in the lungs at the time of death, but they manifested positive SARS-CoV-2 RNA signal in the digestive tract or skin. Intriguingly, these two patients did not show typical COVID-19 pneumonia but died of heart failure caused by dilated cardiomyopathy and infective endocarditis (Case 25) or respiratory failure caused by severe Aspergillus infection (Case 26). Notably, the number of SARS-CoV-2-positive organs was correlated with viral RNA in the lungs (Fig. 1e), suggesting that higher pulmonary viral RNA or deficiency in viral clearance may contribute to the broad virus dissemination. To compare virus-tropic organs between COVID-19 and SARS, we reviewed previous literature regarding virus organotropism.^20–28^ A broader spectrum of organ infection was shown by SARS-CoV-2 as compared to SARS-CoV (Fig. 1f). SARS-CoV-2, but not SARS-CoV, was detected in the aorta, esophagus, thyroid gland, testes and uterus, showing a diffused map of SARS-CoV-2 organotropism in critically ill patients with COVID-19.

### SARS-CoV-2-associated pulmonary pathology

**Fig. 2:**
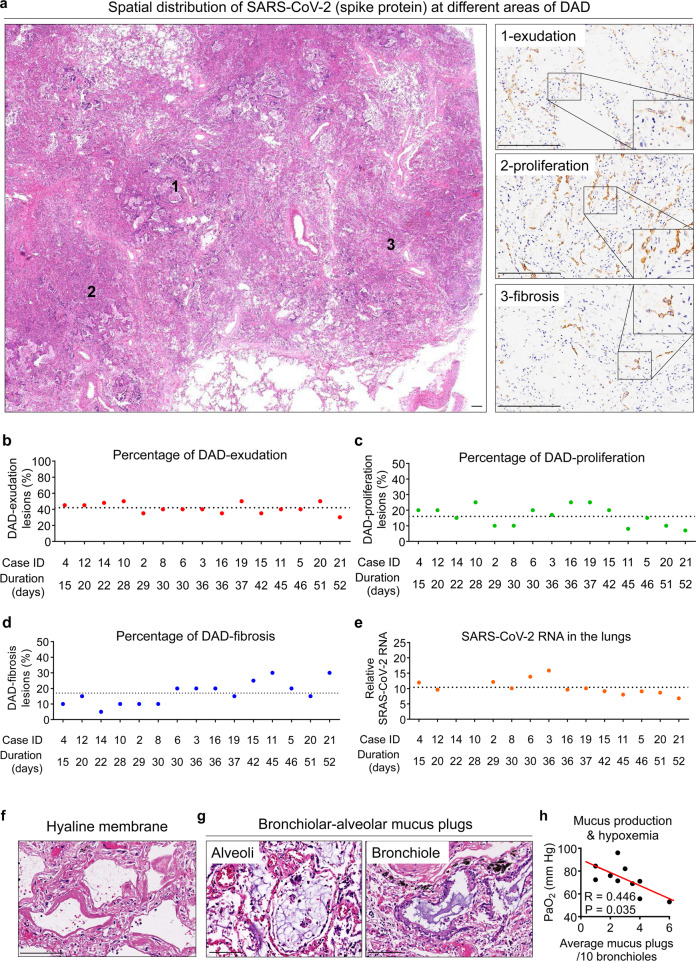
SARS-CoV-2-associated pulmonary pathological changes. **a** H&E and IHC staining showing SARS-CoV-2 spike protein in pulmonary areas manifesting different features (1, exudation; 2, proliferation; 3, fibrosis) of diffuse alveolar damage (DAD). Scale bars, 250 μm. **b**–**e** Proportion of DAD-exudation areas (**b**), DAD-proliferation areas (**c**), and DAD-fibrosis areas (**d**), and the average SARS-CoV-2 RNA (**e**) in postmortem lungs from 15 COVID-19 autopsy cases. **f, g** H&E staining showing hyaline membrane formation (**f**) and bronchiolar-alveolar mucus (**g**). Scale bars, 100 μm. **h** The correlation between average bronchiolar-alveolar mucus plug number and PaO_2_ level in patients with respiratory failure.

Pulmonary pathologies in this cohort (including 6 reported cases from Wuhan Jinyintan Hospital^7^) were featured by three phases of diffuse alveolar damage (DAD): exudation, proliferation, and fibrosis (Fig. 2a; Supplementary information, Fig. S2a).^29^ DAD-exudation phase was presented with pneumocyte injury and exfoliation, serous or fibrinous exudates, and hyaline membrane formation. DAD-proliferation phase was featured by pneumocyte hyperplasia and atypia, as well as macrophage infiltration. DAD-fibrosis phase manifested alveolar fibrosis and/or interstitial fibrosis. SARS-CoV-2-associated pulmonary pathology was quantified in the first 15 consecutive autopsy cases which were tested with SARS-CoV-2 RNA in the lungs. The percentage of DAD-exudation, DAD-proliferation, and DAD-fibrosis areas accounted for 41.5 ± 6.3%, 16.5 ± 6.3%, and 17.0 ± 7.5%, respectively, of total pulmonary tissues in 15 autopsy cases (Fig. 2b–d). Intriguingly, DAD-fibrosis was found as early as 15 days since the symptom onset and increased with disease progression (Fig. 2d), indicating fibrosis as an early and severe COVID-19 complication. Pulmonary areas with higher expression of SARS-CoV-2 spike protein were featured by hyperproliferation of epithelia (Supplementary information, Fig. S2b, c). Further analyses revealed that the proliferative cells containing SARS-CoV-2 were mainly ACE2-expressing and TTF-1-positive alveolar epithelia and bronchiolar basal cells (Supplementary information, Fig. S2d, e). PCR analyses indicated that pulmonary viral RNA maintained a high level from 15–36 days since the symptom onset and showed a gradual decrease with disease duration extension (36–52 days) (Fig. 2e). We also observed extensive pulmonary changes in all postmortem patients with respiratory failure, including alveolar serous and fibrin exudates, hyaline membrane formation, and alveolar/interstitial fibrosis (Fig. 2f; Supplementary information, Fig. S2f), which may form a histopathological basis for impeded air diffusion. Remarkably, in ten COVID-19 patients with medical records of respiratory failure, mechanical ventilation, and arterial oxygen partial pressure (PaO_2_), mucus plugs were present in the alveoli or bronchioles (Fig. 2g), which were inversely associated with the levels of PaO_2_ (Fig. 2h), suggesting that mucus production was increased in hypoxemia and may limit the efficacy of mechanical ventilation during COVID-19 treatment.

### Identification of SARS-CoV-2 in the endothelia of physiological barriers in the lungs, kidneys and testes

**Fig. 3:**
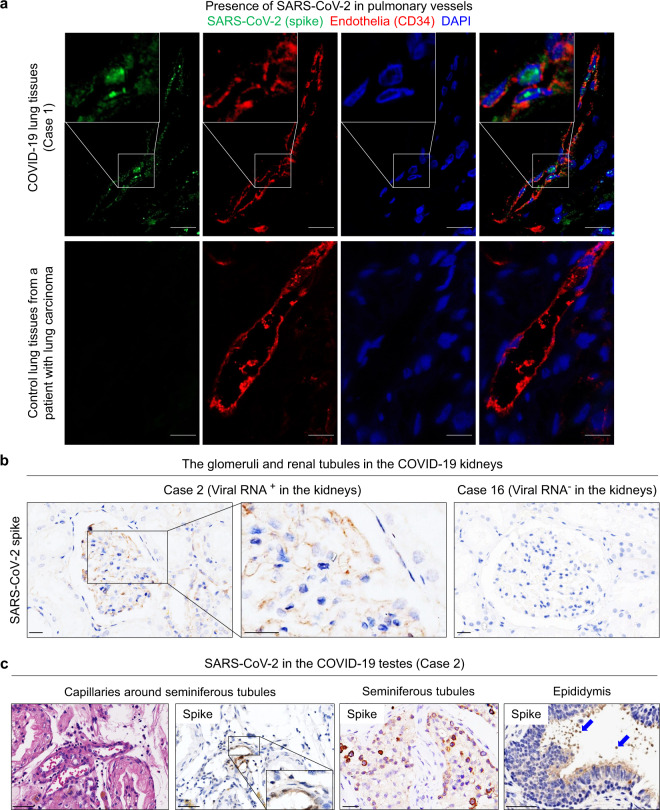
The presence of SARS-CoV-2 in the endothelia of physiological barriers in the lungs, kidneys, and testes. **a** Immunofluorescent staining of SARS-CoV-2 spike protein and CD34 in endothelia of pulmonary vessels using COVID-19 lung tissues (upper panel, Case 1) or control lung tissues from a patient with lung carcinoma (lower panel). Scale bars, 25 μm. **b** IHC showing that SARS-CoV-2 spike protein was detected in glomeruli with abundant filtrated barriers and convoluted tubular epithelia in the kidneys positive for viral RNA (Case 2). The kidney tissues (Case 16) negative for viral RNA were used as control. Scale bars, 25 μm. **c** H&E staining and IHC staining showing SARS-CoV-2 spike in endothelia of the blood–testis barrier, seminiferous tubules, and sperms in the epididymis (blue arrows) of the testes from COVID-19 patients (Case 2). Scale bars, 50 μm.

We found that SARS-CoV-2 spike protein was present in CD34^+^ endothelia at blood–air barrier or pulmonary vessels in serial sections of the COVID-19 lungs (Fig. 3a), raising the possibility that SARS-CoV-2 was able to infiltrate blood–air barrier for intrapulmonary and systemic dissemination. SARS-CoV-2 spike protein was mainly detected in the glomeruli with abundant endothelium-formed filtration barriers and renal proximal convoluted tubular epithelia in the kidneys (Cases 1, 2, 4, and 5) which were positive for SARS-CoV-2 RNA (Fig. 3b), but was not detected in the kidneys (including Case 16) negative for SARS-CoV-2 RNA (Fig. 3b). SARS-CoV-2 spike protein was also detected in endothelia of the blood–testis barrier (Fig. 3c), spermatogenic cells and stromal cells in the seminiferous tubules, and sperms in the epididymis in the COVID-19 testes positive for SARS-CoV-2 RNA (Cases 2, 5 and 11) (Fig. 3c). These results provide evidence of SARS-CoV-2 presence in the endothelia including those in physiological barriers (blood–air, blood–testis, and filtration barriers), implying that these cells are susceptible to SARS-CoV-2 infection followed by systemic dissemination.

### Identification of SARS-CoV-2 in monocytes and macrophages

**Fig. 4:**
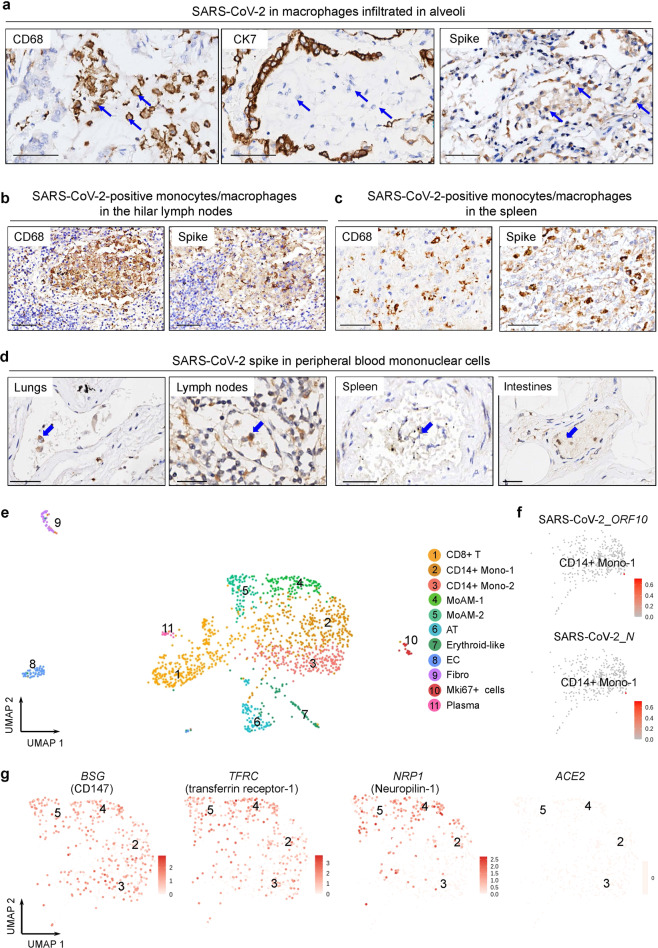
Evidence of the presence of SARS-CoV-2 in circulating and infiltrating monocytes and macrophages. **a** IHC staining of CD68, CK7, and viral spike in alveoli on serial sections. Macrophages are indicated by blue arrows. Scale bars, 50 μm. **b**, **c** IHC staining of monocytes/macrophages marked by CD68 and viral spike protein in lymph nodes (**b**) and the spleen (**c**) on serial sections from COVID-19 patients. Scale bars, 50 μm. **d** IHC staining showing viral spike in peripheral blood mononuclear cells (blue arrows) in vessels of the indicated postmortem organs from COVID-19 patients. Scale bar, 50 μm. **e** U-MAP showing scRNA-seq of 1437 cells on COVID-19 autopsy lung tissues (Case 17). CD8^+^ T, CD8^+^ T cells; CD14^+^ Mono-1/2, CD14^+^ monocyte-1/2; MoAM-1/2, monocyte-derived alveolar macrophages-1/2; AT, alveolar epithelial type 1/2 cells; Erythroid-like, erythroid-like and erythroid precursor cells; EC, endothelial cells; Fibro, fibroblast cells; MKI67^+^, MKI67^+^cells; Plasma, plasma cells. **f** Detection of SARS-CoV-2 transcripts. Plot shows SARS-CoV-2 ORF_10 or nucleocapsid (N) genes in CD14^+^ monocyte-1 from scRNA-seq. **g** U-MAP showing the expression of *BSG* (encoding CD147), *TFRC* (encoding transferrin receptor-1), *NRP1* (encoding neuropilin-1), and *ACE2* in the scRNA-seq of COVID-19 lung tissues.

To investigate the possible routes for viral dissemination, we investigated whether SARS-CoV-2 was contained in monocytes and macrophages, critical immune cell populations.^30^ We found that the cellular components of alveolar exudate were mainly CD68^+^ macrophages positive for SARS-CoV-2 spike protein (Fig. 4a). IHC staining using serial sections also identified the presence of SARS-CoV-2 spike protein in monocytes and macrophages in lymph nodes and the spleen (Fig. 4b, c), as well as peripheral blood mononuclear cells in the postmortem lungs, kidneys, lymph nodes, spleen, and intestines (Fig. 4d). Single-cell RNA-sequencing (scRNA-seq) of lung tissues from a COVID-19 autopsy case (Case 17) within 2 h after death revealed the presence of CD14^+^ monocytes (CD14^+^ Mono-1, -2), monocyte-derived alveolar macrophages (MoAM-1, -2), and other cell types (Fig. 4e; Supplementary information, Fig. S3). CD14^+^ monocytes were characterized by *VCAN* expression and MoAMs were positive for *C1QA* and *C1QC*. Importantly, we detected SARS-CoV-2 transcripts of open reading frame 10 (ORF_10) and nucleocapsid in alveolar CD14^+^ monocyte (Fig. 4f), confirming the presence of SARS-CoV-2 in lung monocytes. To address whether the entry of SARS-CoV-2 into monocytes and macrophages was mediated by receptors other than ACE2, we measured mRNA of previously reported SARS-CoV-2 receptors including *BSG* (encoding CD147), *TFRC* (encoding transferrin receptor-1), and *NRP1* (encoding neuropilin-1) in CD14^+^ monocytes and MoAMs in COVID-19 lungs using scRNA-seq.^31–34^ We found that CD14^+^ Mono-1, -2, and MoAM-1, -2 expressed *BSG*, *TFRC,* and *NRP1*, but not *ACE2* (Fig. 4g), suggesting that CD147, transferrin receptor-1, or neuropilin-1 might mediate SARS-CoV-2 infection of monocytes and macrophages. These findings suggest that SARS-CoV-2 may hijack monocytes and macrophages for systemic dissemination.

## Discussion

Investigations of SARS-CoV-2 organotropism, its duration in the target organs and the correlation with disease progression are pivotal for developing effective strategies for the containment of the pandemic. Some studies revealed SARS-CoV-2 distribution in human tissues and viral shedding in body fluids,^18,35–38^ but the translational significance remains unclear. Our pathological evidence of SARS-CoV-2 presence in late stage of critically ill patients with COVID-19 suggests that continued usage of antiviral therapy is indispensable throughout the disease course. Pulmonary areas with more SARS-CoV-2 exhibit increased diffused alveolar damage and airway obstruction associated with ventilation dysfunction, supporting the cytotoxic effect of SARS-CoV-2 that causes respiratory failure. We demonstrated that the majority of autopsy cases with systemic virus distribution showed multiple organ failures, supporting the systemic nature of the disease. A minor fraction of patients (5/26) with mild virus-related pulmonary damages died of co-morbidities or secondary infections, suggesting complex causes of death related to COVID-19.

While accumulating data support the presence of SARS-CoV-2 in extrapulmonary organs, how the virus systemically spreads remains an enigma. Our study identified SARS-CoV-2 presence in endothelia located at several physiological barriers (blood–air, filtration, and blood–testis barriers), raising the possibility that the virus might invade these barriers for dissemination. The virus was also found in vascular endothelia of multiple organs, which may cause vasculitis.^39^ The injured endothelia may initiate vascular dysfunction and subsequently a procoagulant state to induce thrombosis, as well as ischemic or hemorrhagic changes frequently observed in fatal COVID-19 patients.^40^ Additional cautions should be exercised to treat vulnerable patients with pre-existing diseases associated with high risk of endothelial injury, such as hypertension and diabetes.

Our scRNA-seq of COVID-19 lung tissues, together with previous RNA-seq and tissue staining data, has revealed the presence of SARS-CoV-2 transcripts in CD14^+^ monocytes and macrophages infiltrating the lungs, the spleen, lymph nodes, and the kidneys.^37,41,42^ How monocytes and macrophages became infected remains unclear. Recent scRNA-seq analyses of human tissues have revealed that ACE2 and transmembrane protease serine 2 (TMPRSS2) are rarely expressed in immune cells including monocytes and macrophages.^37^ Our results together with previous studies have revealed that CD147, a recently identified receptor for SARS-CoV-2, was expressed in epithelial cells, lymphocytes, monocytes, and macrophages.^31,32^ The expression of CD147 correlates with the abundance of viral RNA in alveolar macrophages.^37^ Since viremia and viral sepsis in COVID-19 patients have been previously described,^43–45^ the presence of SARS-CoV-2 spike or nucleocapsid protein in monocytes and macrophages may be interpreted as: (1) circulating SARS-CoV-2 directly enters peripheral monocytes or tissue-resident macrophages, as for SARS-CoV and MERS-CoV;^45,46^ (2) circulating monocytes internalize secretory vesicles or cell debris carrying SARS-CoV-2 through endocytosis; (3) tissue-resident macrophages phagocytize virus-infected cells, followed by viral release from lysosome. Also, antibody recognizing the virus may mediate the infection through antibody-dependent enhancement of infection.^47^ As antibody-based therapeutics including anti-SARS-CoV-2 monoclonal antibody and IL-6 receptor antagonist tocilizumab are under clinical evaluation,^48,49^ further investigations of the mechanisms underlying SARS-CoV-2 infection in monocytes and macrophages are urgently warranted.

We and previous studies have identified significant infiltrating monocytes and macrophages in COVID-19 lungs, the spleen, lymph nodes, and the kidneys.^7,14,50,51^ Monocyte and macrophage composition was further characterized by a reduction of tissue-resident alveolar macrophages and an increased abundance of inflammatory monocyte-derived macrophages in critically ill COVID-19 patients.^52^ Increased population of monocytes and macrophages is associated with type I interferon response dysfunction, which has been implicated in increased severity of SARS and MERS similar to findings in COVID-19.^53^ The overloaded monocytes and macrophages may directly interact with interferon-γ-secreting T cells, secrete chemoattractants for immune cell recruitment, or produce pro-inflammatory cytokines to induce alveolitis and subsequent pulmonary damages.^54,55^ Massive pro-inflammatory cytokines released by monocytes and macrophages are associated with respiratory insufficiency and may lead to cytokine storm-associated shock, multiple organ failure, and death in COVID-19 patients.^53^ Pro-inflammatory macrophages may also phagocytize lymphocytes in the spleen and lymph nodes, thus contributing to lymphopenia in COVID-19 patients.^2,30^ In addition, a subset of macrophages harboring tissue repair and fibrosis generation signature has been reported in severe COVID-19 patients, extending the potential pathogenicity of infiltrating macrophages to fibrosis.^56,57^ Therefore, circulating monocytes and tissue infiltrating macrophages are pivotal for systemic and local immune disorders, viral infection, and tissue injuries. Comprehensive evaluations of the signatures of monocytes and macrophages via multimodal single-cell profiling may promote our understanding of the molecular features, activation status, spatial and chronological distribution of monocytes and macrophages in the unique inflammatory milieu of COVID-19, thus benefiting targeted therapeutics.

## Methods

### Patients, autopsy, and clinical data collection

The autopsy cases were from Huoshenshan Hospital (*n* = 8), Taikang Tongji Hospital (*n* = 5), Zhongfaxincheng Hospital (*n* = 5), and Wuhan Jinyintan Hospital (*n* = 8), China. We conducted 26 cases of autopsies from cadaver donors who died from Feb 18th to April 4th, 2020, who had been diagnosed with COVID-19, with written consent from patient’s immediate relatives. Lung tissues from a patient who died from sudden heart death or those from a patient with lung carcinima were used as control tissues for comparison with those from the COVID-19 patients. Fresh or formalin-fixed, paraffin-embedded (FFPE) postmortem specimens were used for viral RNA detection, tissue staining, or morphology analyses. SARS-CoV-2 infection of all cadaver donors was confirmed by virologic tests. Retrospective analyses of case history and clinical manifestations were performed through reviewing the electronic medical records, nursing records, laboratory findings and radiological imaging of the cadaver donors. This study was approved by the ethics committee of Huoshenshan Hospital and is in accordance with regulations issued by the National Health Commission of China and the Helsinki Declaration.

### Tissue staining and transmission electron microscopy

Hematoxylin and eosin (H&E) staining, IHC, and transmission electron microscopy (TEM) were performed according to the standard procedure as described previously.^58,59^ IHC was performed by using the strep-tavidin-biotin-peroxidase technique with diaminobenzidine. Immunofluorescent staining of SARS-CoV-2 and CD34 was performed on FFPE lung sections. Heat-induced antigen epitope retrieval in EDTA (pH 9.0) or citrate buffer (pH 6.0) was applied for optimal detection of antigens on FFPE sections. Sections were incubated overnight at 4 °C with primary antibodies as listed in Supplementary information, Table S1. The specificity and reliability of SARS-CoV-2 spike and nucleoprotein antibody have been verified in previously published literatures^59–61^ and were confirmed by immunocytochemistry staining using in vitro cultured Cercopithecus Vero E6 cells (ATCC^®^ CRL-1586™) transfected with SARS-CoV-2 (Supplementary information, Fig. S4). Vero E6 cells without SARS-CoV-2 were used as control. Staining was visualized by Dako REAL™ EnVision™ Detection System (K5007) followed by counterstaining with hematoxylin. The diluent with control IgG antibodies was used as a negative control. Images were captured by using a digital camera (DP73, Olympus) under a light microscope (BX53, Olympus). The pathological lesions and SARS-CoV-2 spike protein were quantified by two pathologists independently. Briefly, three phases of DAD were evaluated according to the histopathological lesion features. The percentage of each phase of DAD was calculated as the ratio of the pulmonary lesion areas showing histopathological changes of each DAD phase versus the total DAD areas in each slide. For each patient, at least 20 randomly selected 100× microscopic fields were calculated. The percentages of SARS-CoV-2-positive cells in type II pneumocytes and bronchiolar epithelia and Ki67-positive pneumocytes and bronchiolar epithelia in the serial section were quantified under 400× magnification of microscopic fields in at least 10 randomly selected areas for each group.

### Real-time reverse transcription PCR

The nucleic acids of SARS-CoV-2 were detected by real-time reverse transcription PCR method with a SARS-CoV-2 Nucleic Acid Detection Kit (Sansure Biotech) according to the manufacturer’s instructions. The sequences of primers and probes of SARS-CoV-2 were obtained from National Institute for Viral Disease Control and Prevention (http://nmdc.cn/#/nCoV) which were listed in Supplementary information, Table S2. Relative levels of SARS-CoV-2 RNA in tissues were normalized to Rnase P to adjust for differences in tissue input.

### ScRNA-seq and data processing

ScRNA-seq of lung tissues was performed on a COVID-19 autopsy case (Case 17) from whom unfixed lung tissues (right lower lobe presented with focal consolidation) were isolated to single cells within 2 h after tissue collection in a biosafety level-3 (BSL-3) laboratory. Briefly, tissues were digested in GEXSCOPE Tissue Dissociation Solution (Singleron Biotechnologies, Nanjing, China) and were passed through 40-μm nylon sterile strainer to obtain single-cell suspensions. To reduce red blood cells, GEXSCOPE Red Blood Cell Lysis Buffer (Singleron Biotechnologies) was added to the cell suspension. scRNA-seq libraries were constructed following the Singleron’s protocol using a GEXSCOPE Single-cell RNA kit (1110012). After quality checks, scRNA-seq libraries were pooled and sequenced on a NovaSeq 6000 instrument (Illumina) with 150 bp paired end reads. Sequencing outputs were demultiplexed to convert BCL files to FASTQ format using bcl2fastq. Sequencing data were processed using the CeleScope 1.1.7 pipeline (Singleron). To detect viral RNA, reads were aligned to a custom blended genome containing GRCh38.98 and SARS-CoV-2 (ASM985889v3). A viral transcript containing the entire SARS-CoV-2 genome was added to the GRCh38.98GTF files to enable detection of SARS-CoV-2.^55^ After STAR (2.6.1b) alignment and samtools (1.9) filtering,^62,63^ the filtered count matrix was separated by the cell type judgment result obtained by the CeleScope for further analysis. A total of 1551 cells with 1294 genes per cell as median were remained.

### Normalization, clustering and cell type identification of scRNA-seq

Single-nucleus expression matrix by CeleScope was performed using the Seurat package (version3.2.1) for filtering, data normalization, dimensionality reduction, clustering, and gene differential expression analysis.^64^ Briefly, cells with high quality were selected with the following criteria: (1) cells with unique features < 200 or > 5000 were removed; (2) Cells with ≥ 20% mitochondrial counts were removed. For each cell, the counts were log normalized with the “NormalizeData” function. Two thousand variable genes were selected using the “FindVariableGenes” function. A KNN graph based on the euclidean distance was constructed in PCA space. The edge weights between any two cells were refined based on the shared overlap in their local neighborhoods. Cells were clustered using the “FindClusters” function at an appropriate resolution, and were visualized using a Uniform Manifold Approximation and Projection for Dimension Reduction (UMAP) algorithm with the “RunUMAP” function. Wilcoxon rank-sum test was performed for each cluster using the “findMarkers” function to look for differentially expressed genes. Cell type of each cluster was annotated by the known marker genes (Supplementary information, Table S3). Through the above analysis, we processed the scRNA-seq data of 1437 high-quality cells.

### Statistical analysis

All statistical analyses were performed by using SPSS version 13.0 software (SPSS Inc.). Categorical variables were described as frequency rates and percentages, and continuous variables were described using mean, median, or interquartile range (IQR) values. Bivariate correlation analysis (Pearson *r* test) was used to examine the correlation of two variables in human specimens. All data met the assumptions of the tests.

## Supplementary information

Supplementary information, Fig. S1

Supplementary information, Fig. S2

Supplementary information, Fig. S3

Supplementary information, Fig. S4

Supplementary information, Table S1

Supplementary information, Table S2

Supplementary information, Table S3
