# Supplementary material for: A cohort autopsy study defines COVID-19 systemic pathogenesis
Source: Cell Res. 2021 Jun 16;31(8):836–46. doi: 10.1038/s41422-021-00523-8 (PMC8208380; doi:10.1038/s41422-021-00523-8)
Supplement: Supplementary file 1 — Supplementary information, Fig. S1 [file 41422_2021_523_MOESM1_ESM.pdf]

Figure. S1

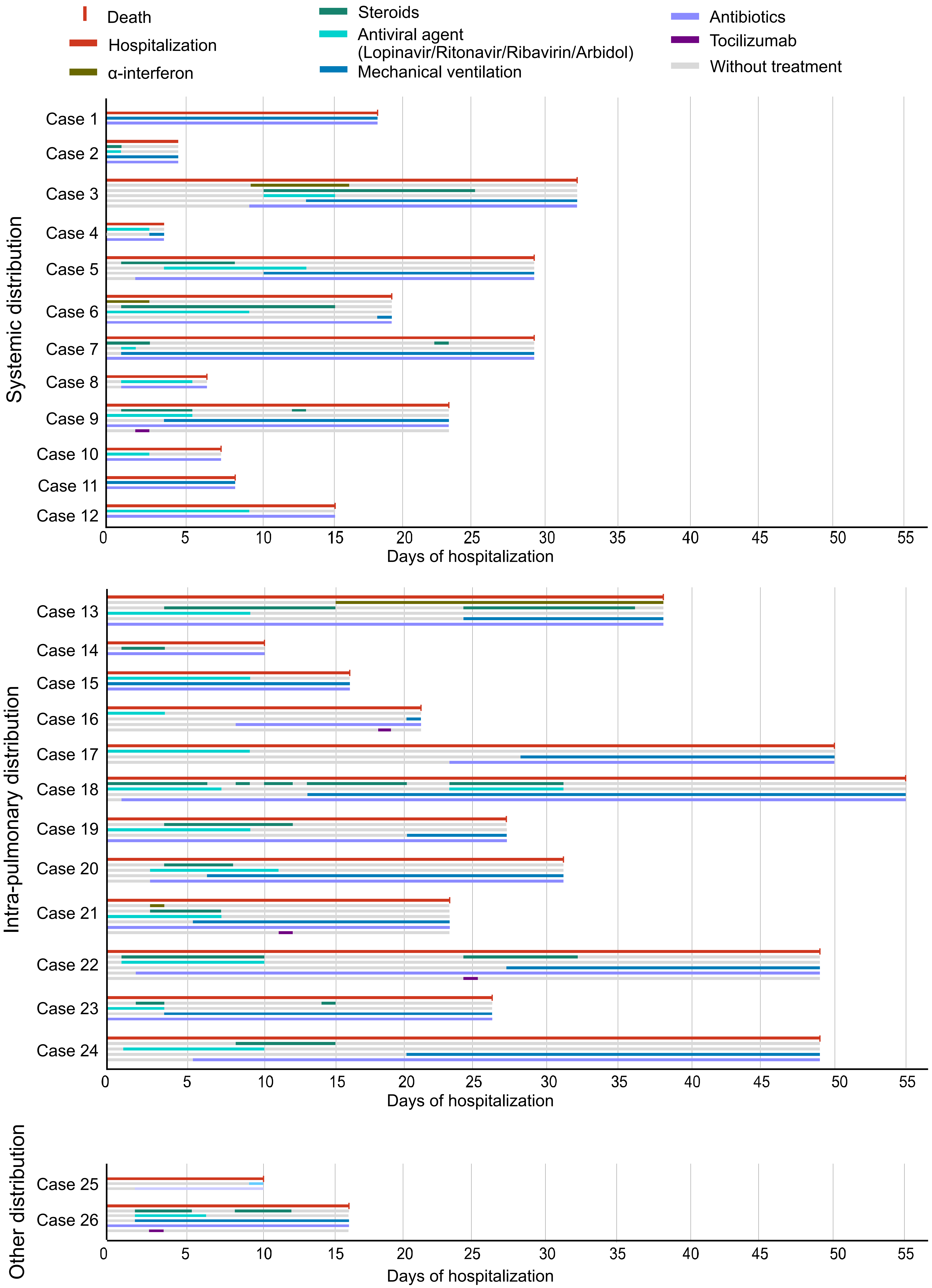

Fig. S1. Treatment regimens of COVID-19 cases in this study. Treatment regimens are indicated in different colors. The short vertical lines indicate the day of patient death.
