# Supplementary material for: A cohort autopsy study defines COVID-19 systemic pathogenesis
Source: Cell Res. 2021 Jun 16;31(8):836–46. doi: 10.1038/s41422-021-00523-8 (PMC8208380; doi:10.1038/s41422-021-00523-8)
Supplement: Supplementary file 2 — Supplementary information, Fig. S2 [file 41422_2021_523_MOESM2_ESM.pdf]

Figure. S2

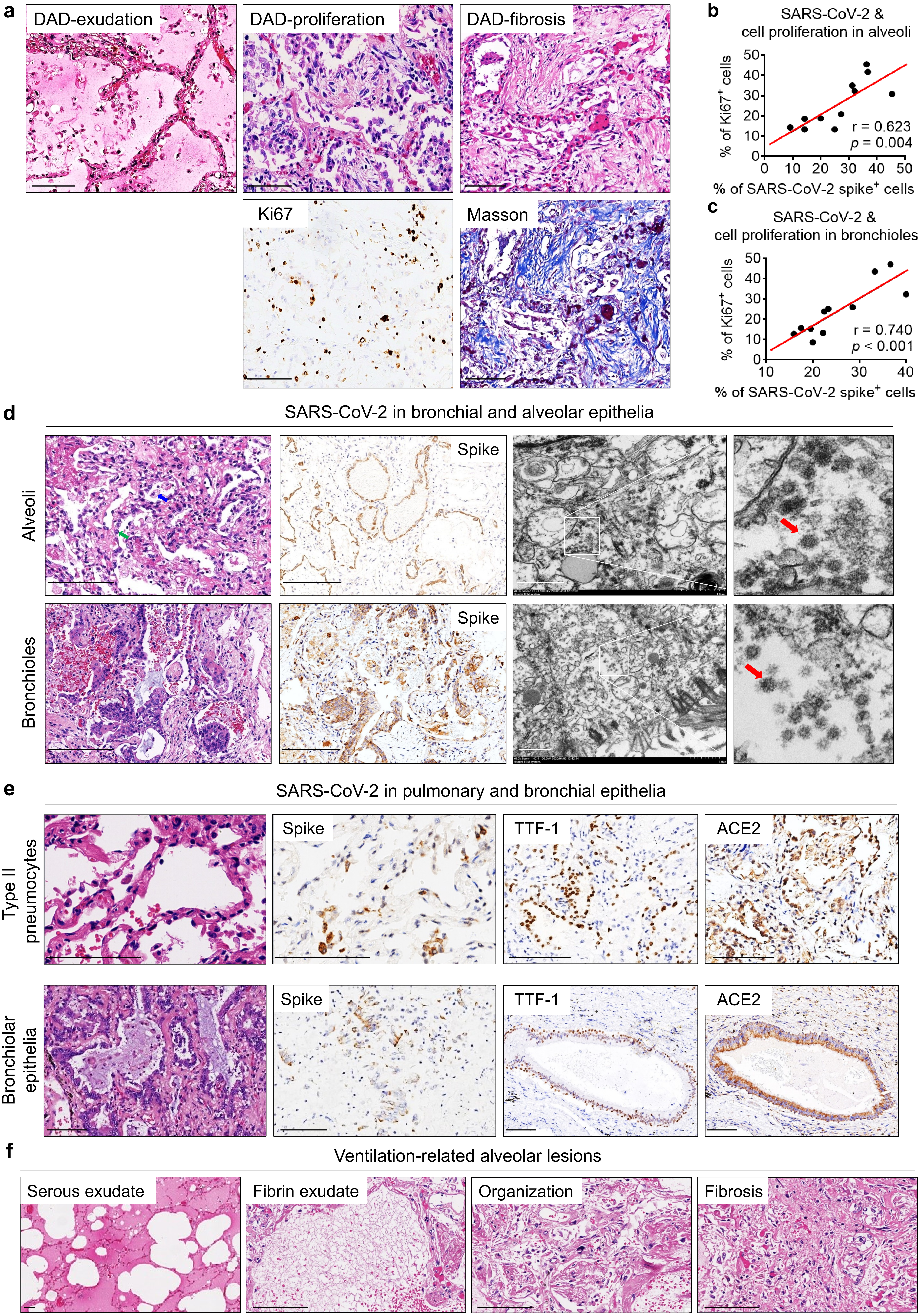
a
DAD-exudation
DAD-proliferation
DAD-fibrosis
Ki67
Masson
b
SARS-CoV-2 &
cell proliferation in alveoli
% of Ki67+ cells
r = 0.623
p = 0.004
% of SARS-CoV-2 spike+ cells
c
SARS-CoV-2 &
cell proliferation in bronchioles
% of Ki67+ cells
r = 0.740
p < 0.001
% of SARS-CoV-2 spike+ cells
d
SARS-CoV-2 in bronchial and alveolar epithelia
Alveoli
Bronchioles
Spike
Spike
e
SARS-CoV-2 in pulmonary and bronchial epithelia
Type II pneumocytes
Bronchiolar epithelia
Spike
TTF-1
ACE2
Spike
TTF-1
ACE2
f
Ventilation-related alveolar lesions
Serous exudate
Fibrin exudate
Organization
Fibrosis

Fig. S2 Pulmonary lesions in COVID-19 patients. a H&E staining, IHC staining of Ki67 (in the middle/lower panel), and Masson staining of collagenous fibers and elastic fibers (blue, in the right/lower panel) showing pathological changes of diffuse alveolar damage (DAD)-exudation, -proliferation and -fibrosis in serial lung sections. Scale bar, 100 μm. **b-c** Correlation of SARS-CoV-2 spike and cell proliferation in alveoli (**b**) and bronchioles (**c**). **d** H&E, IHC staining and TEM detection of SARS-CoV-2 (red arrows) in type II pneumocytes (green arrows), alveolar macrophages (blue arrows) and bronchiolar epithelia. H&E and IHC staining were performed on serial sections. Scale bar, 200 μm for IHC and 1 μm for TEM. **e** H&E and IHC staining of SARS-CoV-2 spike, TTF-1 (a marker of alveolar epithelia and basal cells of distal terminal bronchioles) and ACE2 in alveoli and bronchioles on serial sections. Scale bar, 100 μm. **f** H&E staining showing ventilation-related alveolar lesions as indicated. Scale bar, 100 μm.
