# Supplementary material for: A cohort autopsy study defines COVID-19 systemic pathogenesis
Source: Cell Res. 2021 Jun 16;31(8):836–46. doi: 10.1038/s41422-021-00523-8 (PMC8208380; doi:10.1038/s41422-021-00523-8)
Supplement: Supplementary file 3 — Supplementary information, Fig. S3 [file 41422_2021_523_MOESM3_ESM.pdf]

Figure. S3

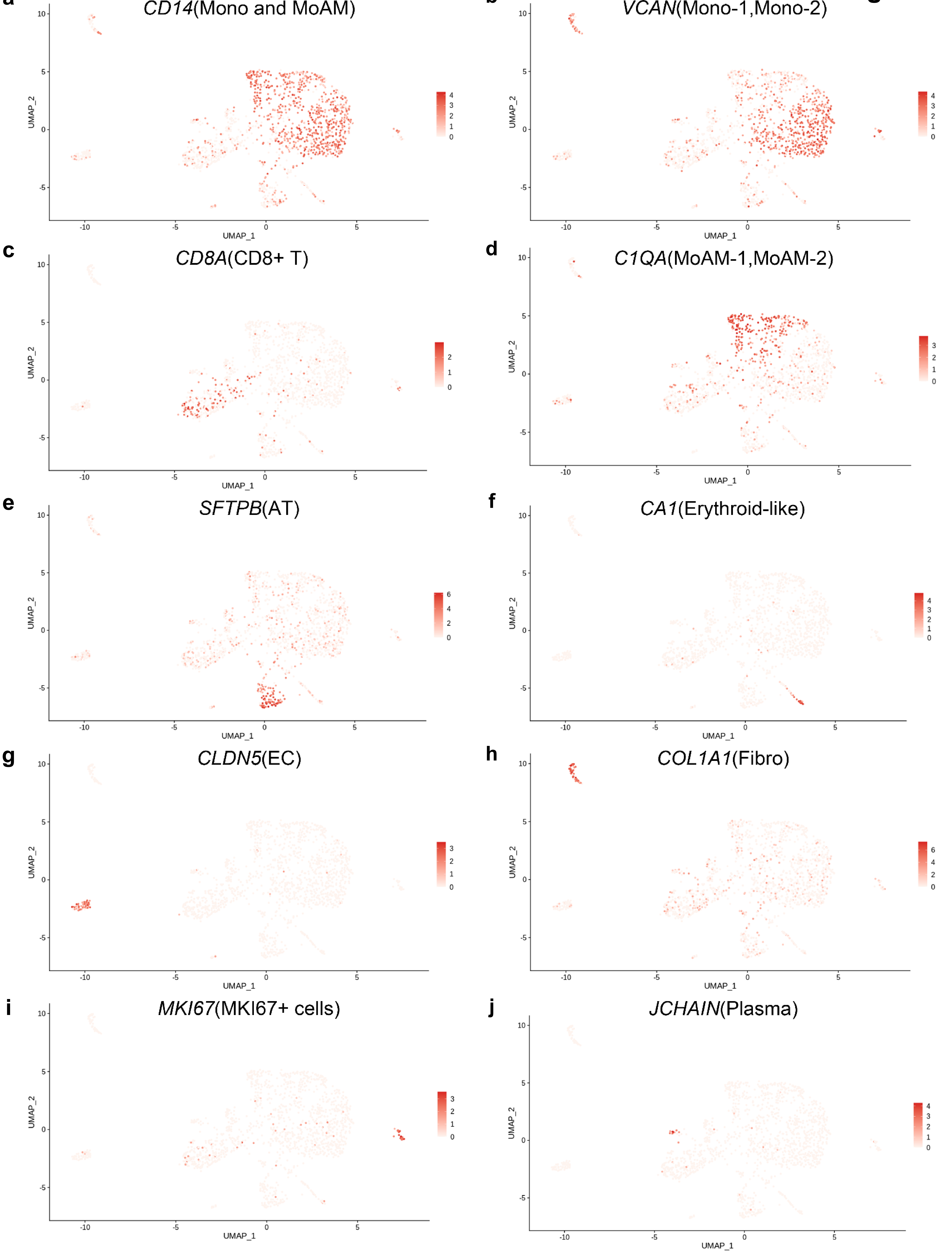

Fig. S3. Markers used for cell type identification in scRNA-seq. **a-j** Known marker genes in the indicated cell subsets. Mono: monocytes; MoAM: monocyte-derived alveolar macrophages; $CD8^+$ T: $CD8^+$ T cells; AT: alveolar epithelial type 1/2 cells; Erythroid-like: erythroid-like and erythroid precursor cells; EC: endothelial cells; Fibro: fibroblast cells; Plasma: plasma cells.
