# Supplementary material for: A cohort autopsy study defines COVID-19 systemic pathogenesis
Source: Cell Res. 2021 Jun 16;31(8):836–46. doi: 10.1038/s41422-021-00523-8 (PMC8208380; doi:10.1038/s41422-021-00523-8)
Supplement: Supplementary file 4 — Supplementary information, Fig. S4 [file 41422_2021_523_MOESM4_ESM.pdf]

Figure. S4

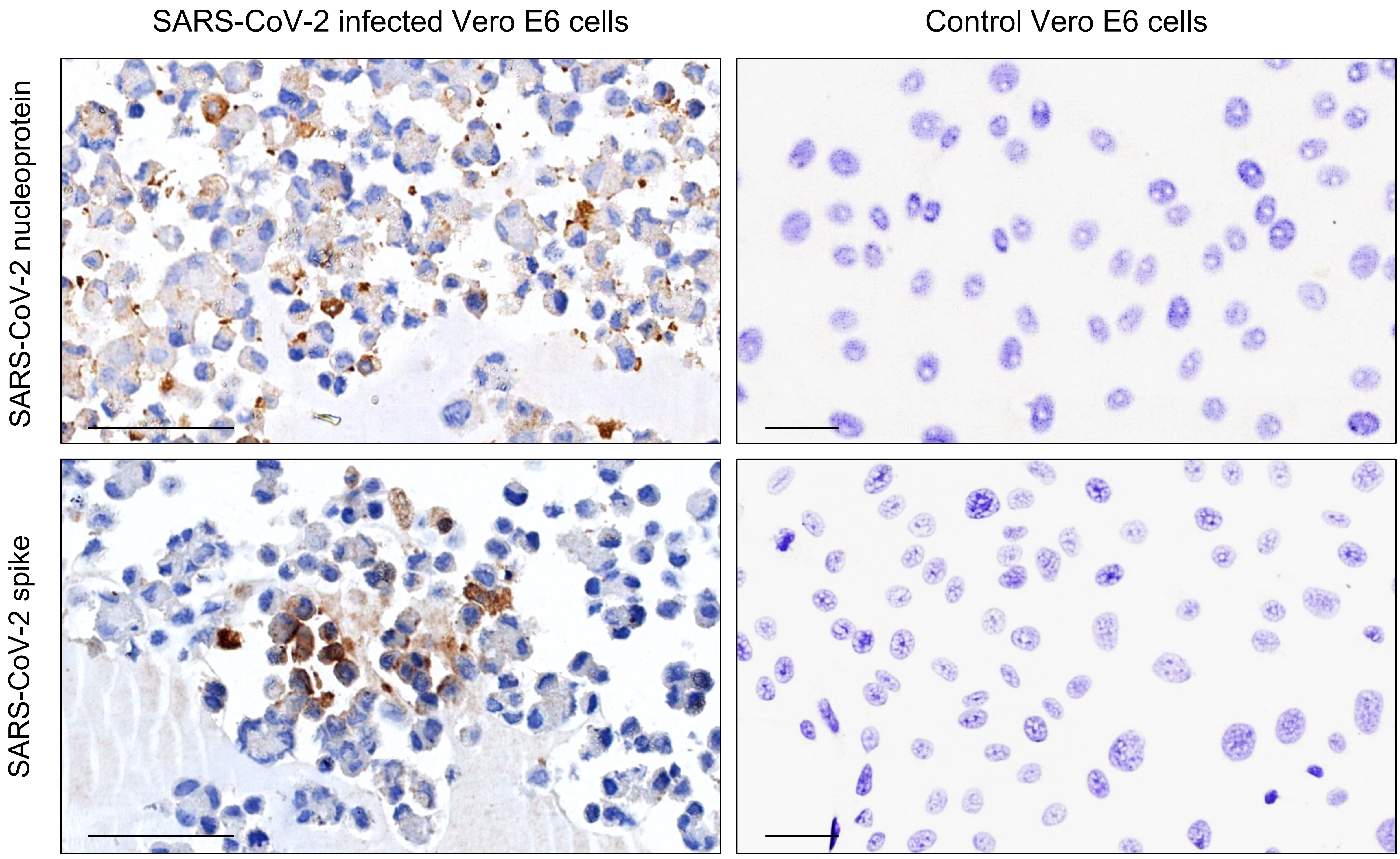

Fig. S4. Immunocytochemical staining of the SARS-CoV-2 nucleoprotein (Sino Biological Inc., 40143-R019) and spike protein (Sino Biological Inc., 40150-T62-COV2) showing the presence of SARS-CoV-2 in *in vitro* cultured Cercopithecus Vero E6 cells with SARS-CoV-2 infection (*left*) but not in control cells (*right*). Scale bar, 50 µm.
