# Supplementary material for: A cohort autopsy study defines COVID-19 systemic pathogenesis
Source: Cell Res. 2021 Jun 16;31(8):836–46. doi: 10.1038/s41422-021-00523-8 (PMC8208380; doi:10.1038/s41422-021-00523-8)
Supplement: Supplementary file 5 — Supplementary information, Table S1 [file 41422_2021_523_MOESM5_ESM.pdf]

Supplementary Tables

Table S1. Primary antibodies used in immunohistochemical staining (IHC) and immunofluorescent staining (IF).

Name	Company	Catalog number	Dilution
SARS-CoV-2 spike	Sino Biological Inc.	40150-T62-COV2	1:1000 for IHC<br>1:100 for IF
SARS-CoV-2 nucleoprotein	Sino Biological Inc.	40143-R019	1:1000
TTF-1	ZSGB-Bio Tech	ZM-0270	ready for use
CD34	MXB Biotechnology	Kit-0004	ready for use
CK7	MXB Biotechnology	Kit-0021	ready for use
CD68	MXB Biotechnology	Kit-0026	ready for use
