# Supplementary material for: A cohort autopsy study defines COVID-19 systemic pathogenesis
Source: Cell Res. 2021 Jun 16;31(8):836–46. doi: 10.1038/s41422-021-00523-8 (PMC8208380; doi:10.1038/s41422-021-00523-8)
Supplement: Supplementary file 6 — Supplementary information, Table S2 [file 41422_2021_523_MOESM6_ESM.pdf]

Table S2. The primers and probes used in RT-PCR.

	Target 1 (open reading frame 1ab, ORF1ab, 5'-3')	Target 2 (nucleoprotein, N, 5'-3')
Forward	CCCTGTGGGTTTTACACTTAA-	GGGGAACTTCTCCTGCTAGAAT
Reverse	ACGATTGTGCATCAGCTGA	CAGACATTTTGCTCTCAAGCTG
Probe	FAM-CCGTCTGCGGTATGTGGAAAGGTTATGG-BHQ1	FAM-TTGCTGCTGCTTGACAGATT-TAMRA
