# Supplementary material for: A cohort autopsy study defines COVID-19 systemic pathogenesis
Source: Cell Res. 2021 Jun 16;31(8):836–46. doi: 10.1038/s41422-021-00523-8 (PMC8208380; doi:10.1038/s41422-021-00523-8)
Supplement: Supplementary file 7 — Supplementary information, Table S3 [file 41422_2021_523_MOESM7_ESM.pdf]

Table S3. Cell type marker genes and differentially expressed genes in scRNA-seq.

p_value	avg_logFC	pct.1	pct.2	p_value_adj	Cell type	Gene Symbol
7.97E-64	6.72E-01	1	0.944	1.59E-59	CD14+ Mono-1	*SH3BGRL3*
1.27E-62	7.29E-01	1	0.854	2.53E-58	CD14+ Mono-1	*IFI30*
1.31E-57	5.54E-01	1	0.999	2.61E-53	CD14+ Mono-1	*FTH1*
2.41E-55	7.29E-01	0.991	0.859	4.81E-51	CD14+ Mono-1	*VIM*
2.47E-52	7.29E-01	0.982	0.799	4.92E-48	CD14+ Mono-1	*S100A10*
2.90E-50	6.75E-01	0.994	0.755	5.77E-46	CD14+ Mono-1	*LGALS1*
7.35E-50	6.94E-01	0.947	0.586	1.46E-45	CD14+ Mono-1	*AIF1*
8.86E-48	9.37E-01	0.93	0.676	1.76E-43	CD14+ Mono-1	*PLIN2*
1.18E-47	6.08E-01	0.994	0.738	2.35E-43	CD14+ Mono-1	*TYROBP*
6.07E-47	7.17E-01	0.982	0.769	1.21E-42	CD14+ Mono-1	*LGALS3*
5.46E-46	5.66E-01	1	0.88	1.09E-41	CD14+ Mono-1	*S100A11*
1.33E-45	7.52E-01	0.833	0.421	2.65E-41	CD14+ Mono-1	*VCAN*
6.60E-44	6.02E-01	0.968	0.658	1.32E-39	CD14+ Mono-1	*FCER1G*
3.18E-43	3.81E-01	1	0.999	6.34E-39	CD14+ Mono-1	*FTL*
4.01E-43	4.31E-01	0.982	0.81	7.99E-39	CD14+ Mono-1	*S100A9*
3.12E-41	4.15E-01	1	0.982	6.22E-37	CD14+ Mono-1	*TMSB10*
3.86E-41	5.98E-01	0.968	0.818	7.69E-37	CD14+ Mono-1	*S100A4*
4.57E-41	4.32E-01	1	0.943	9.11E-37	CD14+ Mono-1	*S100A6*
6.42E-41	6.38E-01	0.915	0.64	1.28E-36	CD14+ Mono-1	*ATP6V1F*
6.58E-40	5.00E-01	0.997	0.941	1.31E-35	CD14+ Mono-1	*GAPDH*
1.86E-37	3.39E-01	0.974	0.791	3.70E-33	CD14+ Mono-1	*S100A8*
6.96E-37	4.71E-01	0.985	0.819	1.39E-32	CD14+ Mono-1	*NPC2*
2.02E-36	4.93E-01	0.997	0.968	4.03E-32	CD14+ Mono-1	*ACTB*
3.09E-35	5.85E-01	0.889	0.611	6.15E-31	CD14+ Mono-1	*GSTO1*
1.06E-34	6.25E-01	0.845	0.484	2.11E-30	CD14+ Mono-1	*VSIG4*
2.72E-34	6.21E-01	0.886	0.509	5.42E-30	CD14+ Mono-1	*CD14*
1.48E-33	4.69E-01	0.994	0.843	2.94E-29	CD14+ Mono-1	*CD63*
1.72E-33	5.50E-01	0.865	0.594	3.42E-29	CD14+ Mono-1	*H2AFY*
3.53E-33	5.58E-01	0.862	0.525	7.04E-29	CD14+ Mono-1	*CTSL*
4.04E-33	6.93E-01	0.962	0.734	8.05E-29	CD14+ Mono-1	*CSTB*
6.87E-60	1.15E+00	0.991	0.805	1.37E-55	CD14+ Mono-2	*S100A8*
6.32E-55	1.17E+00	0.973	0.828	1.26E-50	CD14+ Mono-2	*S100A9*
4.32E-54	1.38E+00	0.773	0.3	8.61E-50	CD14+ Mono-2	*RETN*
6.14E-49	1.07E+00	0.916	0.565	1.22E-44	CD14+ Mono-2	*BCL2A1*
8.49E-48	9.49E-01	0.942	0.621	1.69E-43	CD14+ Mono-2	*AIF1*
2.16E-46	1.05E+00	0.867	0.454	4.31E-42	CD14+ Mono-2	*VCAN*
3.75E-46	8.11E-01	1	0.983	7.48E-42	CD14+ Mono-2	*HSP90AA1*
6.89E-45	8.08E-01	0.996	0.932	1.37E-40	CD14+ Mono-2	*HSPA1A*
1.53E-41	1.15E+00	0.911	0.727	3.05E-37	CD14+ Mono-2	*HSPH1*
1.83E-37	9.24E-01	0.769	0.38	3.64E-33	CD14+ Mono-2	*FPR1*
1.12E-34	7.26E-01	0.947	0.837	2.24E-30	CD14+ Mono-2	*S100A4*
5.31E-33	5.84E-01	0.991	0.965	1.06E-28	CD14+ Mono-2	*UBC*

1.27E-31	6.10E-01	0.969	0.767	2.53E-27	CD14+ Mono-2	*TYROBP*
1.93E-31	7.97E-01	0.747	0.399	3.84E-27	CD14+ Mono-2	*MNDA*
3.62E-31	1.11E+00	0.889	0.731	7.21E-27	CD14+ Mono-2	*HSPD1*
2.03E-29	7.83E-01	0.542	0.215	4.05E-25	CD14+ Mono-2	*FGL2*
3.12E-29	7.06E-01	0.658	0.312	6.22E-25	CD14+ Mono-2	*CSF3R*
8.37E-29	6.74E-01	0.938	0.816	1.67E-24	CD14+ Mono-2	*HSPA1B*
2.85E-28	6.67E-01	0.778	0.424	5.69E-24	CD14+ Mono-2	*MS4A6A*
1.37E-27	7.32E-01	0.684	0.356	2.72E-23	CD14+ Mono-2	*LST1*
3.12E-27	5.11E-01	0.96	0.846	6.22E-23	CD14+ Mono-2	*OAZ1*
3.56E-27	9.78E-01	0.929	0.824	7.10E-23	CD14+ Mono-2	*HSPA5*
1.37E-26	6.02E-01	0.844	0.53	2.73E-22	CD14+ Mono-2	*CYBB*
1.38E-26	6.46E-01	0.804	0.474	2.74E-22	CD14+ Mono-2	*C1orf162*
1.58E-25	7.02E-01	0.92	0.817	3.15E-21	CD14+ Mono-2	*HSPE1*
2.18E-25	7.02E-01	0.444	0.16	4.34E-21	CD14+ Mono-2	*MCEMP1*
7.72E-25	1.19E+00	0.716	0.471	1.54E-20	CD14+ Mono-2	*G0S2*
1.14E-24	9.66E-01	0.733	0.459	2.28E-20	CD14+ Mono-2	*IER3*
1.51E-24	6.80E-01	0.791	0.497	3.00E-20	CD14+ Mono-2	*MAFB*
2.46E-24	6.10E-01	0.871	0.63	4.91E-20	CD14+ Mono-2	*TSPO*
6.18E-129	2.35E+00	0.846	0.254	1.23E-124	CD8+ T	*CD69*
9.24E-103	2.51E+00	0.888	0.471	1.84E-98	CD8+ T	*CCL5*
1.92E-95	1.81E+00	0.674	0.16	3.83E-91	CD8+ T	*TRBC2*
1.78E-94	1.70E+00	0.635	0.13	3.55E-90	CD8+ T	*CD3D*
1.51E-93	1.42E+00	0.556	0.082	3.00E-89	CD8+ T	*CD96*
2.54E-91	9.38E-01	0.992	0.982	5.05E-87	CD8+ T	*RPS27*
2.38E-84	1.85E+00	0.593	0.127	4.74E-80	CD8+ T	*GZMA*
3.34E-81	1.46E+00	0.511	0.08	6.64E-77	CD8+ T	*CD3G*
2.19E-75	1.47E+00	0.551	0.121	4.36E-71	CD8+ T	*CD2*
1.20E-72	1.22E+00	0.452	0.064	2.39E-68	CD8+ T	*SH2D1A*
7.47E-69	1.31E+00	0.663	0.255	1.49E-64	CD8+ T	*CYTIP*
1.16E-66	1.62E+00	0.826	0.443	2.30E-62	CD8+ T	*RGS1*
4.34E-66	1.23E+00	0.427	0.064	8.66E-62	CD8+ T	*TRAC*
1.20E-65	7.43E-01	0.986	0.962	2.40E-61	CD8+ T	*RPL21*
1.22E-65	8.09E-01	0.966	0.953	2.44E-61	CD8+ T	*RPS29*
1.69E-65	1.40E+00	0.851	0.538	3.37E-61	CD8+ T	*CD52*
1.83E-65	1.29E+00	0.441	0.074	3.64E-61	CD8+ T	*KLRK1*
2.07E-65	1.24E+00	0.43	0.066	4.12E-61	CD8+ T	*CD7*
2.87E-63	1.28E+00	0.565	0.167	5.71E-59	CD8+ T	*IL32*
2.84E-62	1.14E+00	0.393	0.054	5.65E-58	CD8+ T	*ITM2A*
6.91E-57	9.53E-01	0.337	0.038	1.38E-52	CD8+ T	*CD3E*
4.13E-56	1.55E+00	0.419	0.081	8.23E-52	CD8+ T	*GZMB*
4.78E-56	1.22E+00	0.539	0.158	9.51E-52	CD8+ T	*CST7*
3.09E-55	1.01E+00	0.924	0.843	6.15E-51	CD8+ T	*BTG1*
5.75E-55	6.68E-01	0.983	0.978	1.15E-50	CD8+ T	*RPL13A*
9.79E-55	1.20E+00	0.486	0.13	1.95E-50	CD8+ T	*ISG20*

3.59E-54	1.57E+00	0.424	0.086	7.16E-50	CD8+ T	*IFNG*
3.53E-52	1.21E+00	0.73	0.423	7.03E-48	CD8+ T	*CXCR4*
1.17E-51	6.74E-01	0.997	0.986	2.33E-47	CD8+ T	*RPL13*
1.91E-51	1.15E+00	0.329	0.044	3.80E-47	CD8+ T	*CD8B*
6.69E-90	1.18E+00	0.558	0.037	1.33E-85	MoAM-1	*LPL*
5.22E-87	1.09E+00	0.717	0.079	1.04E-82	MoAM-1	*NR1H3*
1.52E-73	3.62E-01	0.358	0.012	3.03E-69	MoAM-1	*SPOCD1*
2.64E-70	1.36E+00	0.892	0.206	5.25E-66	MoAM-1	*SDC2*
1.18E-64	1.54E+00	0.983	0.312	2.36E-60	MoAM-1	*ACP5*
1.31E-58	1.08E+00	0.658	0.108	2.61E-54	MoAM-1	*TREM2*
4.51E-57	6.68E-01	0.642	0.097	8.98E-53	MoAM-1	*CYP27A1*
9.05E-57	1.40E+00	0.942	0.33	1.80E-52	MoAM-1	*PLD3*
7.00E-55	1.24E+00	0.95	0.313	1.39E-50	MoAM-1	*GPNMB*
4.88E-51	1.06E+00	1	0.999	9.72E-47	MoAM-1	*FTL*
1.18E-50	1.11E+00	1	0.672	2.35E-46	MoAM-1	*CD68*
4.63E-49	1.07E+00	0.892	0.294	9.23E-45	MoAM-1	*CD9*
2.63E-48	1.43E+00	0.967	0.514	5.24E-44	MoAM-1	*APOE*
3.95E-48	1.11E+00	1	0.999	7.87E-44	MoAM-1	*FTH1*
1.53E-46	1.27E+00	0.908	0.357	3.04E-42	MoAM-1	*LIPA*
1.81E-46	8.02E-01	0.742	0.178	3.60E-42	MoAM-1	*NCEH1*
7.94E-46	1.17E+00	1	0.784	1.58E-41	MoAM-1	*CTSB*
1.61E-45	7.62E-01	0.658	0.137	3.21E-41	MoAM-1	*NRP1*
3.12E-45	5.69E-01	0.567	0.094	6.21E-41	MoAM-1	*SLAMF8*
1.20E-44	1.34E+00	0.925	0.346	2.39E-40	MoAM-1	*APOC1*
1.45E-44	7.13E-01	0.758	0.176	2.89E-40	MoAM-1	*ITGAX*
1.53E-44	1.19E+00	1	0.769	3.04E-40	MoAM-1	*CSTB*
4.15E-44	4.68E-01	0.292	0.021	8.26E-40	MoAM-1	*TM4SF19*
7.56E-43	9.85E-01	0.908	0.32	1.50E-38	MoAM-1	*MSR1*
5.66E-41	1.46E+00	0.958	0.512	1.13E-36	MoAM-1	*FABP5*
3.98E-40	5.84E-01	0.558	0.108	7.93E-36	MoAM-1	*MITF*
7.50E-40	4.29E-01	0.442	0.063	1.49E-35	MoAM-1	*CD109*
7.73E-40	8.43E-01	0.583	0.118	1.54E-35	MoAM-1	*MMP19*
1.56E-39	6.41E-01	0.733	0.191	3.11E-35	MoAM-1	*CXXC5*
2.78E-38	3.54E-01	0.45	0.067	5.53E-34	MoAM-1	*SLC16A10*
1.71E-84	2.33E+00	0.966	0.19	3.41E-80	MoAM-2	*C1QC*
1.83E-79	7.57E-01	0.483	0.024	3.64E-75	MoAM-2	*KCNMA1*
8.11E-79	1.13E+00	0.742	0.082	1.62E-74	MoAM-2	*C2*
1.06E-77	1.32E+00	0.787	0.103	2.11E-73	MoAM-2	*SLCO2B1*
2.05E-73	1.80E+00	0.966	0.207	4.09E-69	MoAM-2	*HLA-DQA1*
3.25E-72	1.61E+00	0.809	0.122	6.47E-68	MoAM-2	*PLTP*
3.11E-66	1.93E+00	0.966	0.255	6.20E-62	MoAM-2	*C1QA*
5.88E-66	1.55E+00	0.91	0.188	1.17E-61	MoAM-2	*NUPR1*
9.13E-66	2.06E+00	0.978	0.261	1.82E-61	MoAM-2	*C1QB*
4.54E-57	1.72E+00	0.989	0.325	9.05E-53	MoAM-2	*GPNMB*

5.49E-55	9.37E-01	0.652	0.093	1.09E-50	MoAM-2	*FPR3*
9.64E-55	1.78E+00	1	0.406	1.92E-50	MoAM-2	*HLA-DQB1*
7.73E-54	5.27E-01	0.371	0.023	1.54E-49	MoAM-2	*CMKLR1*
4.61E-53	2.30E+00	0.989	0.523	9.18E-49	MoAM-2	*APOE*
3.13E-52	1.78E+00	1	0.505	6.24E-48	MoAM-2	*HLA-DPB1*
4.63E-52	2.49E+00	0.831	0.212	9.22E-48	MoAM-2	*CCL18*
8.16E-52	1.07E+00	0.596	0.081	1.63E-47	MoAM-2	*PMP22*
2.06E-51	1.70E+00	1	0.932	4.10E-47	MoAM-2	*CD74*
4.98E-51	1.49E+00	0.978	0.342	9.93E-47	MoAM-2	*PLD3*
2.82E-50	1.81E+00	0.978	0.356	5.61E-46	MoAM-2	*APOC1*
5.02E-50	6.76E-01	0.393	0.031	1.00E-45	MoAM-2	*HLA-DOA*
5.37E-50	1.73E+00	1	0.792	1.07E-45	MoAM-2	*HLA-DRA*
6.13E-50	1.25E+00	0.809	0.191	1.22E-45	MoAM-2	*MS4A4A*
1.23E-49	6.23E-01	0.348	0.022	2.46E-45	MoAM-2	*LILRB5*
2.40E-48	1.54E+00	0.989	0.478	4.78E-44	MoAM-2	*HLA-DPA1*
3.48E-48	1.49E+00	0.955	0.329	6.94E-44	MoAM-2	*ACP5*
4.28E-47	1.02E+00	0.82	0.195	8.53E-43	MoAM-2	*CPVL*
5.50E-46	1.68E+00	1	0.728	1.10E-41	MoAM-2	*HLA-DRB1*
8.20E-46	1.21E+00	0.809	0.2	1.63E-41	MoAM-2	*IFITM10*
1.10E-45	9.85E-01	0.685	0.129	2.19E-41	MoAM-2	*TMEM176A*
1.55E-175	1.50E+00	0.744	0.014	3.08E-171	AT	*EPCAM*
1.78E-152	2.66E+00	0.919	0.054	3.55E-148	AT	*KRT7*
1.09E-145	1.38E+00	0.558	0.006	2.17E-141	AT	*AQP5*
6.60E-145	1.84E+00	0.756	0.028	1.32E-140	AT	*TMC5*
6.19E-142	1.64E+00	0.651	0.016	1.23E-137	AT	*CXCL17*
9.26E-140	2.08E+00	0.698	0.023	1.84E-135	AT	*SLC34A2*
4.24E-139	1.17E+00	0.581	0.01	8.44E-135	AT	*MAL2*
2.03E-137	1.43E+00	0.581	0.01	4.05E-133	AT	*FOLR1*
2.83E-132	9.73E-01	0.535	0.007	5.65E-128	AT	*EHF*
1.00E-131	2.07E+00	0.686	0.026	1.99E-127	AT	*CEACAM6*
1.30E-131	1.26E+00	0.547	0.009	2.58E-127	AT	*AGR3*
2.61E-131	9.29E-01	0.523	0.007	5.19E-127	AT	*S100A14*
8.45E-131	1.54E+00	0.663	0.022	1.68E-126	AT	*FXYD3*
3.08E-126	2.50E+00	0.756	0.041	6.14E-122	AT	*PIGR*
3.19E-126	1.44E+00	0.709	0.031	6.35E-122	AT	*KRT8*
3.69E-126	1.27E+00	0.512	0.007	7.34E-122	AT	*KLK11*
1.99E-125	2.59E+00	0.779	0.046	3.96E-121	AT	*AGR2*
2.74E-123	3.19E+00	0.942	0.093	5.47E-119	AT	*WFDC2*
2.82E-123	1.69E+00	0.767	0.044	5.62E-119	AT	*MUC1*
3.09E-119	1.69E+00	0.547	0.013	6.17E-115	AT	*CP*
2.47E-118	1.97E+00	0.837	0.064	4.93E-114	AT	*KRT18*
1.34E-115	1.32E+00	0.593	0.02	2.67E-111	AT	*TACSTD2*
6.59E-115	1.20E+00	0.57	0.017	1.31E-110	AT	*ELF3*
1.45E-114	7.68E-01	0.43	0.004	2.89E-110	AT	*ADH1C*

9.33E-113	1.09E+00	0.628	0.025	1.86E-108	AT	*PERP*
2.60E-112	9.78E-01	0.5	0.01	5.18E-108	AT	*SCNN1A*
5.34E-108	7.25E-01	0.419	0.004	1.06E-103	AT	*SLC44A4*
1.16E-106	5.86E-01	0.395	0.003	2.32E-102	AT	*RAB25*
1.74E-105	1.01E+00	0.477	0.01	3.48E-101	AT	*CD24*
1.46E-103	3.39E+00	0.953	0.127	2.90E-99	AT	*LCN2*
4.64E-67	9.79E-01	0.291	0.005	9.24E-63	Erythroid-like	*SLC4A1*
8.00E-53	2.58E+00	0.316	0.014	1.59E-48	Erythroid-like	*CA1*
5.30E-47	1.48E+00	0.266	0.01	1.05E-42	Erythroid-like	*AHSP*
1.75E-35	3.09E+00	0.342	0.034	3.49E-31	Erythroid-like	*HBD*
7.88E-33	7.60E-01	0.316	0.031	1.57E-28	Erythroid-like	*SNCA*
1.31E-27	4.42E+00	1	0.878	2.61E-23	Erythroid-like	*HBB*
3.14E-27	1.81E+00	0.772	0.35	6.26E-23	Erythroid-like	*SLC25A37*
4.05E-27	4.23E+00	0.949	0.769	8.07E-23	Erythroid-like	*HBA2*
6.43E-22	1.38E+00	0.532	0.168	1.28E-17	Erythroid-like	*SLC25A39*
1.34E-20	4.27E+00	0.823	0.619	2.67E-16	Erythroid-like	*HBA1*
3.67E-09	7.47E-01	0.494	0.247	7.30E-05	Erythroid-like	*GYPC*
5.00E-08	5.14E-01	0.367	0.144	9.96E-04	Erythroid-like	*EPB41*
5.51E-203	1.51E+00	0.702	0.002	1.10E-198	EC	*ROBO4*
4.60E-200	1.82E+00	0.772	0.007	9.17E-196	EC	*TM4SF18*
2.74E-184	2.05E+00	0.754	0.009	5.45E-180	EC	*CLDN5*
1.43E-175	3.01E+00	0.93	0.027	2.85E-171	EC	*MMRN1*
1.60E-173	2.08E+00	0.754	0.011	3.19E-169	EC	*ECSCR*
2.16E-173	1.45E+00	0.667	0.005	4.30E-169	EC	*RAMP2*
2.80E-160	1.69E+00	0.807	0.019	5.58E-156	EC	*CALCRL*
3.68E-158	1.54E+00	0.684	0.009	7.33E-154	EC	*VWF*
6.49E-158	1.46E+00	0.702	0.011	1.29E-153	EC	*PCAT19*
9.71E-156	2.60E+00	0.807	0.021	1.93E-151	EC	*APOD*
2.54E-155	1.23E+00	0.579	0.004	5.07E-151	EC	*DIPK2B*
3.11E-155	2.75E+00	0.93	0.036	6.20E-151	EC	*GNG11*
3.21E-153	1.65E+00	0.702	0.012	6.40E-149	EC	*EGFL7*
4.64E-151	2.74E+00	0.702	0.013	9.24E-147	EC	*ANGPT2*
6.60E-151	1.37E+00	0.614	0.007	1.31E-146	EC	*PROX1*
1.52E-148	1.09E+00	0.632	0.008	3.03E-144	EC	*CDH5*
5.54E-148	9.37E-01	0.526	0.002	1.10E-143	EC	*MYCT1*
8.97E-146	1.27E+00	0.632	0.009	1.79E-141	EC	*AFAP1L1*
1.41E-140	1.16E+00	0.544	0.004	2.82E-136	EC	*LDB2*
3.83E-139	2.91E+00	0.825	0.03	7.64E-135	EC	*AKAP12*
5.92E-139	1.75E+00	0.772	0.023	1.18E-134	EC	*COL4A1*
2.73E-138	9.01E-01	0.561	0.006	5.44E-134	EC	*RHOJ*
3.15E-136	1.04E+00	0.474	0.001	6.28E-132	EC	*CD34*
6.13E-136	8.19E-01	0.474	0.001	1.22E-131	EC	*SEMA6A*
1.37E-135	1.93E+00	0.737	0.02	2.73E-131	EC	*TFF3*
2.10E-134	1.65E+00	0.719	0.02	4.18E-130	EC	*SEMA3A*

6.06E-132	2.23E+00	0.947	0.051	1.21E-127	EC	*TM4SF1*
2.08E-131	1.55E+00	0.737	0.022	4.13E-127	EC	*COL4A2*
4.36E-131	1.85E+00	0.754	0.025	8.68E-127	EC	*ARHGAP29*
6.06E-130	1.87E+00	0.772	0.027	1.21E-125	EC	*CAVIN2*
4.05E-190	1.80E+00	0.744	0.005	8.08E-186	Fibro	*COL14A1*
6.37E-171	2.39E+00	0.884	0.017	1.27E-166	Fibro	*COL5A1*
7.81E-168	1.45E+00	0.581	0.001	1.56E-163	Fibro	*THBS2*
4.95E-162	2.21E+00	0.93	0.024	9.85E-158	Fibro	*COL5A2*
3.61E-160	1.99E+00	0.884	0.02	7.18E-156	Fibro	*TPM2*
1.60E-158	2.98E+00	0.953	0.027	3.19E-154	Fibro	*COL6A3*
2.01E-156	2.62E+00	0.907	0.024	4.01E-152	Fibro	*COL6A1*
7.40E-151	1.40E+00	0.628	0.006	1.47E-146	Fibro	*MFAP4*
5.89E-149	1.21E+00	0.558	0.003	1.17E-144	Fibro	*LINC00632*
2.20E-148	1.76E+00	0.674	0.009	4.38E-144	Fibro	*FGF7*
7.37E-148	1.69E+00	0.674	0.009	1.47E-143	Fibro	*FBLN1*
3.48E-145	8.88E-01	0.488	0.001	6.93E-141	Fibro	*MOXD1*
4.14E-144	1.70E+00	0.837	0.021	8.25E-140	Fibro	*BGN*
3.79E-143	1.12E+00	0.558	0.004	7.55E-139	Fibro	*MXRA5*
7.39E-141	1.46E+00	0.581	0.005	1.47E-136	Fibro	*PRRX1*
1.02E-136	1.60E+00	0.721	0.014	2.03E-132	Fibro	*PCOLCE*
9.24E-136	1.41E+00	0.767	0.018	1.84E-131	Fibro	*FSTL1*
4.01E-134	3.02E+00	0.907	0.032	7.98E-130	Fibro	*LUM*
4.53E-132	1.95E+00	0.651	0.011	9.02E-128	Fibro	*ADH1B*
2.00E-130	7.48E-01	0.419	0	3.99E-126	Fibro	*PODN*
2.94E-129	1.08E+00	0.512	0.004	5.86E-125	Fibro	*PDGFRA*
7.61E-129	1.73E+00	0.767	0.02	1.52E-124	Fibro	*CDH11*
9.20E-129	9.94E-01	0.558	0.006	1.83E-124	Fibro	*CD248*
1.42E-128	8.17E-01	0.512	0.004	2.83E-124	Fibro	*SPON1*
1.91E-125	1.27E+00	0.558	0.006	3.80E-121	Fibro	*LAMA2*
4.05E-125	1.13E+00	0.558	0.006	8.07E-121	Fibro	*ITGBL1*
3.05E-120	1.09E+00	0.512	0.005	6.08E-116	Fibro	*COL8A1*
2.99E-118	8.90E-01	0.442	0.002	5.95E-114	Fibro	*FAP*
4.05E-118	1.55E+00	0.558	0.008	8.07E-114	Fibro	*COL12A1*
1.08E-116	6.67E-01	0.419	0.001	2.15E-112	Fibro	*MMP23B*
1.28E-150	1.16E+00	0.708	0.006	2.55E-146	MKI67+ cells	*PCLAF*
6.41E-142	1.14E+00	0.583	0.003	1.28E-137	MKI67+ cells	*KIF2C*
1.07E-137	1.39E+00	0.792	0.011	2.14E-133	MKI67+ cells	*BUB1B*
3.73E-128	1.15E+00	0.583	0.004	7.42E-124	MKI67+ cells	*UBE2C*
1.52E-113	2.66E+00	1	0.032	3.03E-109	MKI67+ cells	*MKI67*
7.06E-113	1.05E+00	0.458	0.002	1.41E-108	MKI67+ cells	*RRM2*
7.55E-113	1.99E+00	0.792	0.016	1.50E-108	MKI67+ cells	*ASPM*
8.63E-111	1.30E+00	0.667	0.01	1.72E-106	MKI67+ cells	*KIF11*
2.01E-104	8.76E-01	0.542	0.006	3.99E-100	MKI67+ cells	*GTSE1*
3.67E-104	1.04E+00	0.5	0.004	7.32E-100	MKI67+ cells	*DLGAP5*

3.74E-93	7.54E-01	0.333	0.001	7.46E-89	MKI67+ cells	*HJURP*
1.72E-92	9.37E-01	0.458	0.004	3.43E-88	MKI67+ cells	*CKAP2L*
3.68E-89	1.87E+00	0.875	0.031	7.32E-85	MKI67+ cells	*TOP2A*
1.42E-87	8.35E-01	0.458	0.005	2.83E-83	MKI67+ cells	*BIRC5*
1.20E-86	6.92E-01	0.417	0.004	2.39E-82	MKI67+ cells	*MELK*
2.91E-81	7.15E-01	0.375	0.003	5.80E-77	MKI67+ cells	*AURKB*
1.40E-76	1.12E+00	0.583	0.013	2.79E-72	MKI67+ cells	*TPX2*
2.36E-76	9.12E-01	0.417	0.005	4.71E-72	MKI67+ cells	*KIF15*
2.70E-76	6.04E-01	0.333	0.002	5.38E-72	MKI67+ cells	*CDCA5*
1.71E-71	4.92E-01	0.292	0.001	3.41E-67	MKI67+ cells	*PKMYT1*
1.41E-70	8.38E-01	0.375	0.004	2.81E-66	MKI67+ cells	*TK1*
6.37E-65	1.68E+00	0.75	0.033	1.27E-60	MKI67+ cells	*CENPF*
7.67E-62	9.57E-01	0.5	0.013	1.53E-57	MKI67+ cells	*CDK1*
9.61E-62	7.35E-01	0.417	0.008	1.92E-57	MKI67+ cells	*SHCBP1*
1.27E-61	7.65E-01	0.417	0.008	2.53E-57	MKI67+ cells	*CEP55*
2.26E-61	1.99E+00	0.917	0.059	4.50E-57	MKI67+ cells	*NUSAP1*
7.07E-61	1.40E+00	0.833	0.045	1.41E-56	MKI67+ cells	*PRC1*
8.88E-60	1.01E+00	0.5	0.013	1.77E-55	MKI67+ cells	*NUF2*
4.45E-57	6.09E-01	0.458	0.011	8.86E-53	MKI67+ cells	*BRCA1*
1.61E-56	1.73E+00	0.75	0.039	3.21E-52	MKI67+ cells	*HIST1H1B*
5.11E-173	1.41E+00	0.765	0.004	1.02E-168	Plasma	*DERL3*
6.71E-136	2.83E+00	1	0.016	1.34E-131	Plasma	*MZB1*
9.79E-117	2.70E+00	0.765	0.01	1.95E-112	Plasma	*JCHAIN*
2.33E-111	6.89E-01	0.353	0	4.64E-107	Plasma	*FCRL5*
1.61E-100	5.81E-01	0.412	0.001	3.20E-96	Plasma	*TNFRSF17*
2.15E-96	1.12E+00	0.647	0.008	4.28E-92	Plasma	*IGHG2*
2.48E-95	8.31E-01	0.353	0.001	4.95E-91	Plasma	*AC104699.1*
2.48E-95	4.34E-01	0.353	0.001	4.95E-91	Plasma	*AC012236.1*
4.70E-93	6.30E-01	0.294	0	9.35E-89	Plasma	*PNOC*
4.70E-93	4.75E-01	0.294	0	9.35E-89	Plasma	*DPEP1*
1.53E-82	7.94E-01	0.412	0.003	3.05E-78	Plasma	*SPAG4*
1.29E-77	5.82E-01	0.294	0.001	2.57E-73	Plasma	*IGLV6-57*
3.99E-75	6.60E-01	0.412	0.004	7.95E-71	Plasma	*CD79A*
4.31E-57	5.03E+00	0.941	0.051	8.58E-53	Plasma	*IGHG3*
1.29E-55	7.70E-01	0.353	0.004	2.58E-51	Plasma	*JSRP1*
2.02E-52	1.69E+00	0.588	0.018	4.02E-48	Plasma	*IGLC1*
3.49E-51	9.89E-01	0.471	0.011	6.94E-47	Plasma	*MEF2B*
7.96E-45	2.24E+00	0.353	0.006	1.59E-40	Plasma	*IGHV3-7*
7.94E-37	4.95E-01	0.353	0.008	1.58E-32	Plasma	*PAOX*
9.11E-35	5.72E+00	1	0.115	1.82E-30	Plasma	*IGHG4*
3.46E-27	5.99E+00	1	0.157	6.89E-23	Plasma	*IGHG1*
4.79E-27	3.26E+00	0.412	0.019	9.54E-23	Plasma	*IGHM*
9.24E-22	9.05E-01	0.529	0.041	1.84E-17	Plasma	*COBLL1*
3.75E-21	6.87E-01	0.471	0.032	7.48E-17	Plasma	*SDC1*

5.14E-20	1.40E+00	0.706	0.085	1.02E-15	Plasma	*FKBP11*
7.85E-17	6.65E-01	0.353	0.023	1.56E-12	Plasma	*CD79B*
1.76E-16	5.53E-01	0.529	0.052	3.50E-12	Plasma	*ADAM28*
2.05E-16	5.68E-01	0.412	0.032	4.08E-12	Plasma	*RALGPS2*
4.21E-16	5.76E+00	0.941	0.275	8.38E-12	Plasma	*IGKC*
2.44E-15	5.76E-01	0.353	0.026	4.87E-11	Plasma	*CHPF*

Abbreviation: Mono: monocytes; MoAM: monocyte-derived alveolar macrophages; AT: alveolar epithelial type 1/2 cells; Erythroid-like: erythroid-like and erythroid precursor cells; EC: endothelial cells; Fibro: fibroblast cells; Plasma: plasma cells
